# Psychotropic and pain medication use in individuals with traumatic brain injury—a Swedish total population cohort study of 240 000 persons

**DOI:** 10.1136/jnnp-2020-324353

**Published:** 2021-02-09

**Authors:** Yasmina Molero, David James Sharp, Brian Matthew D'Onofrio, Henrik Larsson, Seena Fazel

**Affiliations:** 1 Clinical Neuroscience, Center for Psychiatry Research, Karolinska Institute, Stockholm, Sweden; 2 Psychiatry, University of Oxford, Oxford, UK; 3 Medical Epidemiology and Biostatistics, Karolinska Institute, Stockholm, Sweden; 4 Brain Sciences, Imperial College London, London, UK; 5 Psychological and Brain Sciences, Indiana University Bloomington, Bloomington, Indiana, USA; 6 School of Medical Sciences, Örebro Universitet, Orebro, Sweden

## Abstract

**Objective:**

To examine psychotropic and pain medication use in a population-based cohort of individuals with traumatic brain injury (TBI), and compare them with controls from similar backgrounds.

**Methods:**

We assessed Swedish nationwide registers to include all individuals diagnosed with incident TBI between 2006 and 2012 in hospitals or specialist outpatient care. Full siblings never diagnosed with TBI acted as controls. We examined dispensed prescriptions for psychotropic and pain medications for the 12 months before and after the TBI.

**Results:**

We identified 239 425 individuals with incident TBI, and 199 658 unaffected sibling controls. In the TBI cohort, 36.6% had collected at least one prescription for a psychotropic or pain medication in the 12 months before the TBI. In the 12 months after, medication use increased to 45.0%, an absolute rate increase of 8.4% (p<0.001). The largest post-TBI increases were found for opioids (from 16.3% to 21.6%, p<0.001), and non-opioid pain medications (from 20.3% to 26.6%, p<0.001). The majority of prescriptions were short-term; 20.6% of those prescribed opioids and 37.3% of those with benzodiazepines collected prescriptions for more than 6 months. Increased odds of any psychotropic or pain medication were associated with individuals before (OR: 1.62, 95% CI: 1.59 to 1.65), and after the TBI (OR: 2.30, 95% CI: 2.26 to 2.34) as compared with sibling controls, and ORs were consistently increased for all medication classes.

**Conclusion:**

High rates of psychotropic and pain medications after a TBI suggest that medical follow-up should be routine and review medication use.

## Introduction

Traumatic brain injury (TBI) is an injury to the head from an external force that disrupts brain function. It varies in severity from a mild concussion to severe injury—where there is an extended loss of consciousness and long-term disability. TBI is a global health problem that is estimated to affect 30–60 million persons^1^ and cost $400 billion per year.^2^

TBI significantly increases the risk of physical illness, mortality and neuropsychiatric problems, including epilepsy, depression, anxiety, cognitive disorders and behavioural changes (often aggression or agitation).^2^ ^3^ ^4^ Psychotropic medications are often prescribed as part of post-TBI treatment, as they target symptoms that can have a considerable effect on health and daily life, including depression, anxiety, and, rarely, psychosis.^5^ Previous studies have shown high rates of psychotropic medication use in individuals who have sustained a TBI, but estimates vary widely. For example, prescriptions range between 14% and 74% for antiepileptic medications, 8%–82% for antidepressants, 10%–68% for antipsychotic medications and 6%–33% for anxiolytics.^6–12^ The varying estimates could be attributed to differences in TBI severity and samples; TBI inclusion in studies has ranged from mild^7^ to moderate to severe,^8 9 11^ and samples have included both hospitalised patients,^10^ individuals in outpatient postacute treatment,^8 12^ and those in inpatient rehabilitation.^6 7 9 11^ Results show that the highest rates of antiepileptic (47%–74%), antidepressant (67%–82%) and anxiolytic medications (15%–33%) were found among individuals who received inpatient rehabilitation,^6 7 11^ while patients with moderate to severe TBI or having inpatient treatment were prescribed antipsychotics at the upper end (48%–68%) of the reported ranges.^8 11^ Studies also show that polypharmacy (ie, the use of several medications concurrently) is common.^6–9^ However, previous investigations have been small, typically included selected samples, and have not used reliable sources of prescription information.^6–11^ Importantly, studies have generally not assessed pre-TBI medication use, which is a major confound, as psychiatric disorders and behavioural problems are more common in TBI patients,^13–15^ and would lead to higher rates of background psychotropic medications. One prior study has examined pre-TBI mediation use,^10^ but only included persons older than 64 years.

Many TBI patients experience pain,^16^ and headaches are the most common complaint after a TBI.^5^ The pain can be a consequence of the TBI and/or other physical injuries co-occurring with the TBI.^17^ Pain medications are therefore commonly prescribed to TBI patients, but there is little data on the extent and duration of their use,^18^ particularly for opioid medications. The limited information available is mainly based on US military service members and veterans,^19^ with a different age structure, comorbid painful conditions and sociodemographic and background health risks to those who sustain TBIs in the general population.^16^ Prolonged use of pain medications, particularly opioids, is problematic in other contexts,^20^ therefore, a detailed investigation of opioid prescription after TBI is warranted. Previous research has also not included a matched comparison group, which is important for assessing whether and how medication use among persons with TBI differs from the general population.

To address the uncertainty on psychotropic and pain medication use, we have investigated a total population cohort of people who have sustained TBI. We have compared medication use with unaffected siblings. Such siblings provide a more valid comparison group than unrelated population controls, as they have similar background characteristics (eg, family history and shared genetic and environmental factors)^21^ which can lead to residual confounding.

### Aim

We investigated the prevalence of the main psychotropic and pain medication classes in a longitudinal cohort of adults (aged 18 and over) diagnosed with new incident TBI. We examined dispensed prescriptions (ie, collected at a pharmacy) for the 12 months before and after the incident TBI, which were compared with unaffected full siblings. We assessed the duration of use for each medication class, and also stratified analyses by factors that may confound medication use in TBI patients, including comorbid neuropsychiatric diagnoses and injury severity.

## Methods

### Design

This is a population-based longitudinal cohort study in Sweden linking registers with nationwide coverage through each individual’s identification number.^22^ All data were pseudonymised.

### Participants and setting

Participants included all individuals aged 18 and over diagnosed with new incident TBI diagnosis between 1 July 2006 and 31 December 2012. We used the Centers for Disease Control and Prevention definition of TBI.^23^ We also included unaffected siblings as a comparison group to account for similar background factors (eg, genetics and early environment). Full details are provided in the online supplemental appendix (p2).

10.1136/jnnp-2020-324353.supp1 Supplementary data

### Measures

#### Demographic measures

Information on sex and age was collected from the Total Population Register.^22^

#### Polytrauma

Polytrauma was defined as an injury to another body part or system on the same day as the TBI (ICD-10: S00-S99, T00-T19, T90-T98, excluding TBI diagnoses).

#### Psychotropic and pain medications

Information on medications was collected from the Swedish Prescribed Drug Register, and included opioids, non-opioid pain medications, antiepileptic medications, antipsychotic medications, benzodiazepines, selective serotonin reuptake inhibitors (SSRIs), other antidepressants and attention-deficit hyperactivity disorder (ADHD) medications (full details are provided in the online supplemental appendix, p2).

#### Neuropsychiatric disorders

Information on neuropsychiatric disorders was collected from the Swedish Patient Register, and included International Classification of Diseases, 10th revision [ICD-10] diagnoses recorded during admissions to hospitals and outpatient contacts with specialised secondary care, including substance use disorders (F10–F19), psychotic disorders (F20–F29), mood disorders (F30–F34, F38–F39), anxiety disorders (F40–F45, F48), ADHD (F90) and seizures (G40–G41, R56).

### Statistical analyses

For neuropsychiatric diagnoses, proportions are presented for 12 months before and after the incident TBI for the TBI cohort, and for unaffected full sibling controls.

For each individual in the TBI cohort, medication periods were divided into 12 months before, and 12 months after, the incident TBI. We calculated the proportion of individuals in the TBI cohort with any psychotropic and/or pain medication for the 12 months before and after the incident TBI. We also calculated the proportion of individuals with each specific medication class, the proportion of individuals with only one medication class, and two or more different medication classes.

We also compared medication use 12 months before and after the incident TBI in those with TBIs to their unaffected full siblings. In these analyses, we included a subsample of individuals (n=1 14 314) who had at least one unaffected full sibling as control (n=1 99 658; 60.3% of the TBI cohort had two or more siblings in the control group). To compare medication rates, each sibling control was assigned the same date as their TBI sibling counterpart, and medication periods were divided into 12 months before, and 12 months after, the date of the TBI. For sibling controls with two or more siblings with TBI, the first TBI date of their TBI sibling counterparts was assigned. Individuals with TBI were considered exposed and unaffected siblings were considered unexposed. We applied a fixed-effects model using conditional logistic regression to compare exposed and unexposed individuals on each medication class, where each family was considered a stratum. By design, these models control for shared familial confounders such as genetics and early environmental factors.^24^ Results are expressed as odds ratios (ORs) with 95% confidence intervals (CIs).

In further analyses, we stratified medication use by neuropsychiatric diagnoses received within 12 months of the incident TBI, and calculated the proportion of individuals in each diagnostic category with psychotropic and/or pain medications. We then examined the duration of usage for prescriptions that were initiated within 12 months after the incident TBI (full details are provided in the online supplemental appendix, p3).

### Sensitivity analyses

We carried out sensitivity analyses where we investigated associations between injury severity and medication use using logistic regression models. In these analyses, we compared: (1) individuals who received inpatient treatment (ie, hospitalisation) to individuals who received outpatient treatment (ie, specialised secondary care) for the incident TBI; (2) individuals with moderate, severe and other TBIs to individuals with mild TBI (ie, concussions; ICD-10: S06.0) and (3) individuals with polytrauma (ie, TBI and at least one co-occurring physical injury) to individuals with TBI without co-occurring physical injuries.

## Results

### Characteristics of the TBI cohort and sibling controls

We identified 239 425 individuals aged 18 and over, diagnosed with new incident TBI diagnosis in hospital or specialist outpatient care between 1 July 2006 and 31 December 2012 (table 1). Mean age at the incident TBI was 52 years (SD 24), and the cohort was 58.9% (n=140 893) male. In the TBI cohort, 28.6% (n=68 381) presented with a mild TBI, and 27.0% (n=64 777) were hospitalised as inpatients. The most common premorbid neuropsychiatric diagnoses were substance use (3.3%), mood (2.8%) and anxiety (2.5%) disorders. Absolute rates of neuropsychiatric diagnoses 12 months after the incident TBI increased between 0.1% (for ADHD) and 2.9% (for substance use disorders).

**Table 1:** Demographic and neuropsychiatric characteristics of the traumatic brain injury (TBI) cohort, and in unaffected full siblings

	TBI cohort (n=239 425)	Siblings (n=199 658)
Sex		
Males	58.9% (140 893)	49.1% (98 055)
Females	41.2% (98 532)	50.9% (101 603)
Age at incident TBI*		
18–24	18.4% (43 939)	15.1% (30 232)
25–34	12.9% (30 910)	17.1% (34 123)
35–44	11.7% (27 993)	15.5% (31 035)
45–54	11.6% (27 778)	17.7% (35 274)
55–64	12.0% (28 701)	19.3% (38 617)
65 and older	33.5% (80 104)	15.2% (30 377)
Mean (SD) age at incident TBI	52 (24)	45 (17)
Incident TBI characteristics		
Inpatient treatment	27.0% (64 777)	–
Outpatient treatment	73.0% (174 648)	–
Mild TBI	28.6% (68 381)	–
All other TBI	71.4% (171 044)	–
Polytrauma	18.6% (44 509)	–
Neuropsychiatric diagnoses 12 months before incident TBI*		
Substance use disorders	3.3% (7888)	0.7% (1302)
Psychotic disorders	0.6% (1396)	0.4% (798)
Mood disorders	2.8% (6619)	1.3% (2575)
Anxiety disorders	2.5% (5987)	1.2% (2436)
ADHD	0.6% (1331)	0.2% (485)
Seizures	1.5% (3641)	0.4% (757)
Neuropsychiatric diagnoses 12 months after incident TBI*		
Substance use disorders	6.2% (14 889)	0.7% (1425)
Psychotic disorders	0.7% (1652)	0.4% (781)
Mood disorders	3.4% (8033)	1.4% (2721)
Anxiety disorders	3.0% (7216)	1.4% (2707)
ADHD	0.7% (1677)	0.3% (567)
Seizures	2.7% (6334)	0.4% (767)

*For unaffected full siblings, age and 12-month prevalence of neuropsychiatric diagnoses were calculated from the date of their sibling’s incident TBI.

ADHD, attention-deficit hyperactivity disorder.

We identified 199 658 unaffected sibling controls (defined as full siblings who had not been diagnosed with TBI before the end of the study period) (table 1). Of these, 49.1% (n=98 055) were males, and mean age at the date of their sibling’s TBI was 45 years (SD 17). Sibling controls presented with a lower prevalence of diagnosed neuropsychiatric disorders both before and after their sibling's TBI; mood and anxiety disorders were most common, followed by substance use disorders.

### Psychotropic and pain medication preincident and postincident TBI

**Figure 1:**
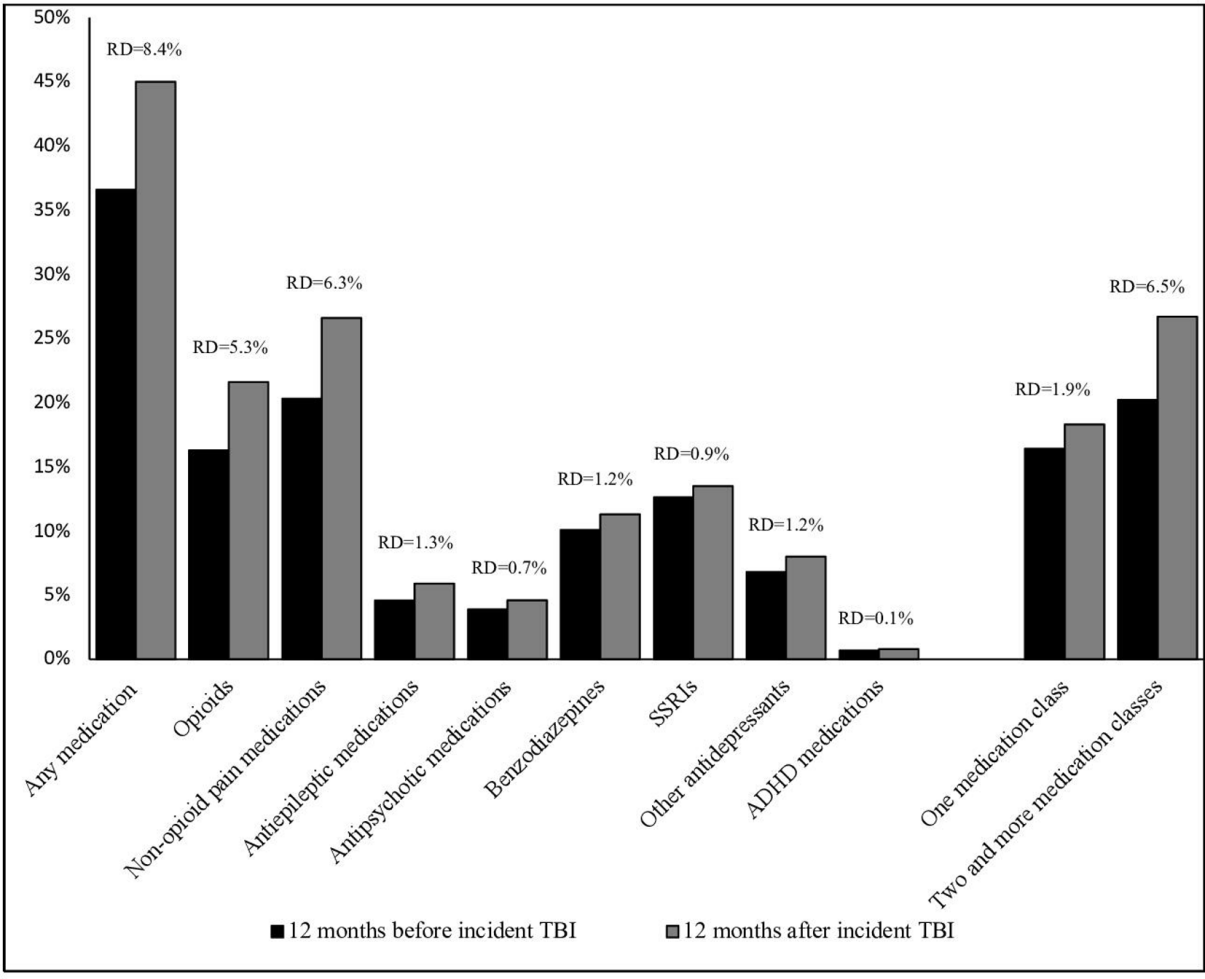
Psychotropic and pain medications 12 months before and 12 months after incident traumatic brain injury (TBI) (n=239 425). All rate differences were associated with p<0.001. *χ* ^2^ and degrees of freedom for each comparison are provided in online supplemental appendix table 1. RD, Rate differences; ADHD, attention-deficit hyperactivity disorder; SSRIs, selective serotonin reuptake inhibitors.

In the 12 months before the incident TBI, 36.6% (n=87 625) of the cohort had collected a prescription for at least one psychotropic or pain medication (figure 1; statistical analyses in online supplemental appendix table 1). In the 12 months after the incident TBI, there was an 8.4% increase in absolute rates of psychotropic medication use to 45.0% (*χ* ^2^ [1]=81.4). There was also an increase in the use of two or more different medication classes within 12 months—from 20.2% pre-TBI to 26.7% post-TBI (*χ* ^2^ [4]=111.7). Pain medications were the most common classes; opioids were prescribed to 16.3% persons in the 12 months before the TBI, and to 21.6% in the 12 months after, an increase in absolute rates of 5.3% (*χ* ^2^ ([1]=46.1). Non-opioid pain medications increased by 6.3%; from 20.3% to 26.6% (*χ* ^2^ ([1]=67.8). Absolute rates after the TBI increased by around 1% for all psychotropic medications except for ADHD medications (which increased from 0.7% to 0.8%). All pre-TBI and post-TBI differences in medication use were statistically significant (p<0.001).

### Psychotropic and pain medication in TBI cohort compared with sibling controls

**Figure 2:**
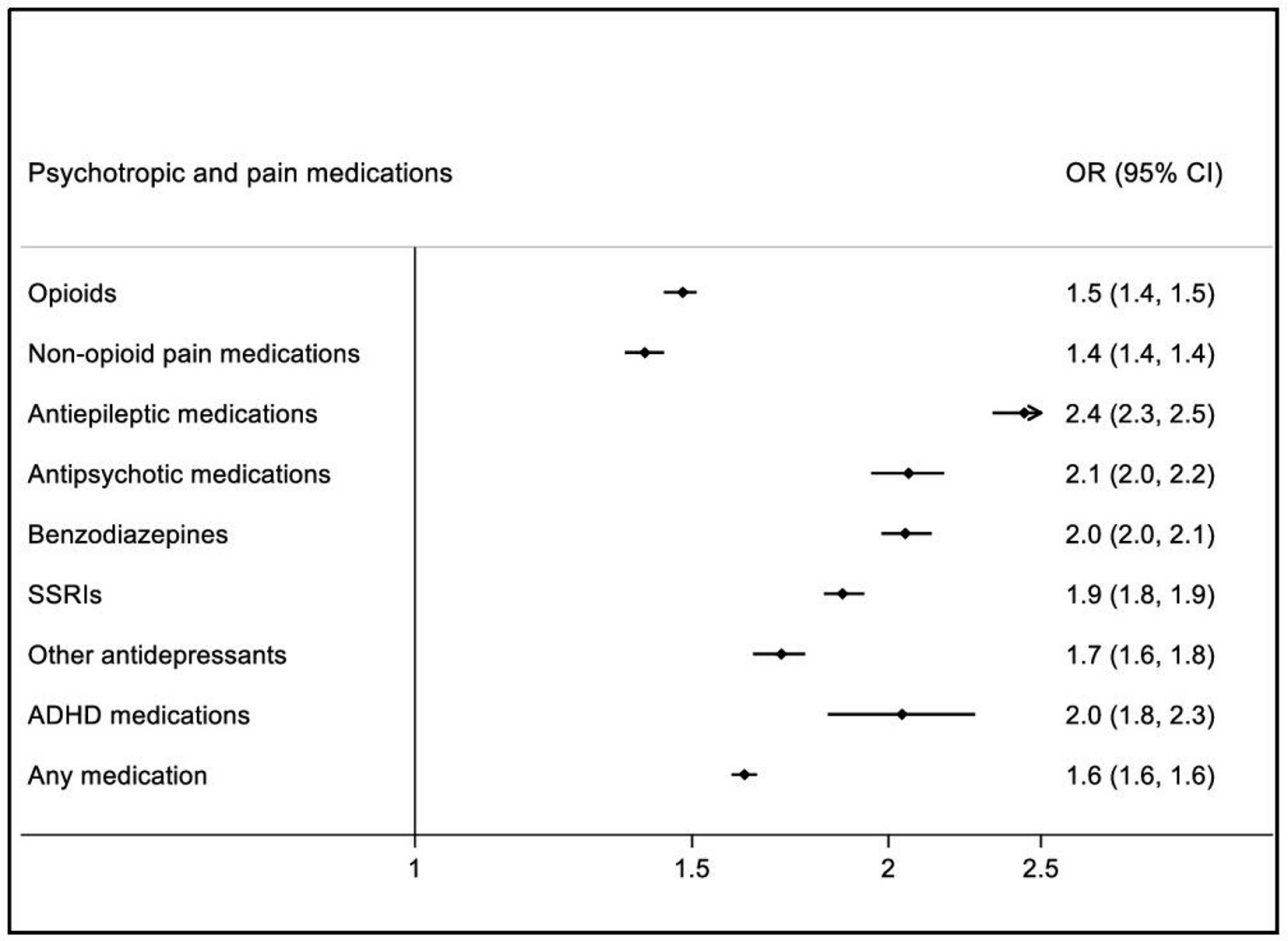
ORs of being prescribed psychotropic and pain medications in TBI cohort (n=114 314)† compared with their unaffected full siblings (n=199 658) during 12 months before the TBI. †Including only TBI individuals with at least one full unaffected sibling. Note: All ORs were associated with p<0.0001. ADHD, attention-deficit hyperactivity disorder; SSRIs, selective serotonin reuptake inhibitors.

**Figure 3:**
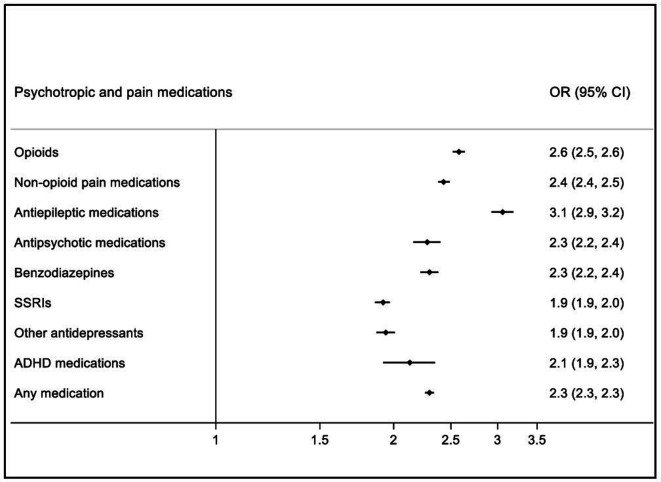
ORs of being prescribed psychotropic and pain medications in TBI cohort (n=114 314)† compared with their unaffected full siblings (n=199 658) during 12 months after the TBI. †Including only TBI individuals with at least one full unaffected sibling. Note: All ORs were associated with p <0.0001. ADHD, attention-deficit hyperactivity disorder; SSRIs, selective serotonin reuptake inhibitors.

We then compared medication use in the TBI cohort, both before and after the TBI, with that in unaffected full sibling controls in the same time period. This comparison included 114 314 TBI cases (who had at least one full unaffected sibling), and 199 658 sibling controls. In the 12 months before the incident TBI (figure 2; prevalence rates in online supplemental appendix table 2), individuals in the TBI cohort had higher odds of collecting a psychotropic and/or pain medication prescription when compared with their siblings (OR: 1.62, 95% CI: 1.59 to 1.65). Individuals in the TBI cohort were more likely to collect prescriptions for all medication classes, with the largest differences for antiepileptic (OR: 2.44, 95% CI: 2.33 to 2.55) and antipsychotic medications (OR: 2.06, 95% CI: 1.95 to 2.17), and benzodiazepines (OR: 2.05, 95% CI: 1.98 to 2.13). In the 12 months after the incident TBI (figure 3; prevalence rates in online supplemental appendix table 2), the odds of collecting a prescription for at least one psychotropic and/or pain medication increased in the TBI cohort (OR: 2.30, 95% CI: 2.26 to 2.34). Individuals in the TBI cohort presented with higher odds of all medication classes when compared with their siblings after the TBI, with the largest increases for antiepileptic medications (OR: 3.06, 95% CI: 2.93 to 3.19), opioids (OR: 2.58, 95% CI: 2.52 to 2.64) and non-opioid pain medications (OR: 2.43, 95% CI: 2.38 to 2.49).

### Psychotropic and pain medications by post-TBI neuropsychiatric diagnosis

We stratified medication use by neuropsychiatric diagnoses received within 12 months of the incident TBI (table 2). Common medications among individuals diagnosed with substance use disorders were opioid and non-opioid pain medications (prescribed to around 25%) and benzodiazepines (prescribed to 21.7%). The most commonly prescribed medication among individuals diagnosed with psychotic disorders were antipsychotic medications (81.0%), followed by benzodiazepines (41.7%). Among individuals with mood or anxiety disorders, antidepressants and benzodiazepines were prescribed to between 40.5% and 54.2%. Results also showed that 35% of individuals with mood or anxiety disorders were prescribed an opioid, and 35%–40% were prescribed a non-opioid pain medication. For individuals with ADHD or seizures, the most common medications were ADHD medications (65.6%) and antiepileptics (75.2%), respectively.

**Table 2:** Psychotropic and pain medications prescribed during the 12 months after incident traumatic brain injury (TBI), stratified by post-TBI neuropsychiatric diagnoses

	Substance use disorders (n=14 889)	Psychotic disorders (n=1652)	Mood disorders (n=8033)	Anxiety disorders (n=7216)	ADHD (n=1677)	Seizures (n=6334)
Opioids	25.8% (3847)	22.2% (366)	35.7% (2866)	35.5% (2545)	25.9% (435)	28.9% (1830)
Non-opioid pain medications	24.8% (3699)	30.7% (507)	39.4% (3163)	34.6% (2,496)	30.7% (507)	41.0% (2594)
Antiepileptic medications	15.9% (2370)	25.2% (417)	26.2% (2107)	24.1% (1736)	21.8% (366)	75.2% (4764)
Antipsychotic medications	11.8% (1752)	81.0% (1338)	30.2% (2424)	21.1% (1525)	22.7% (381)	11.1% (704)
Benzodiazepines	21.7% (3235)	41.7% (689)	40.5% (3250)	43.1% (3113)	26.3% (441)	34.0% (2155)
SSRIs	21.1% (3146)	28.0% (463)	54.2% (4356)	46.2% (3330)	28.3% (475)	23.1% (1464)
Other antidepressants	18.4% (2743)	21.0% (345)	49.6% (3983)	41.8% (3013)	26.2% (438)	14.7% (933)
ADHD medications	3.4% (510)	2.7% (45)	4.3% (342)	5.8% (418)	65.6% (1101)	1.3% (85)

ADHD, attention-deficit hyperactivity disorder; SSRIs, selective serotonin reuptake inhibitors.

### Duration of psychotropic and pain medication prescription

**Figure 4:**
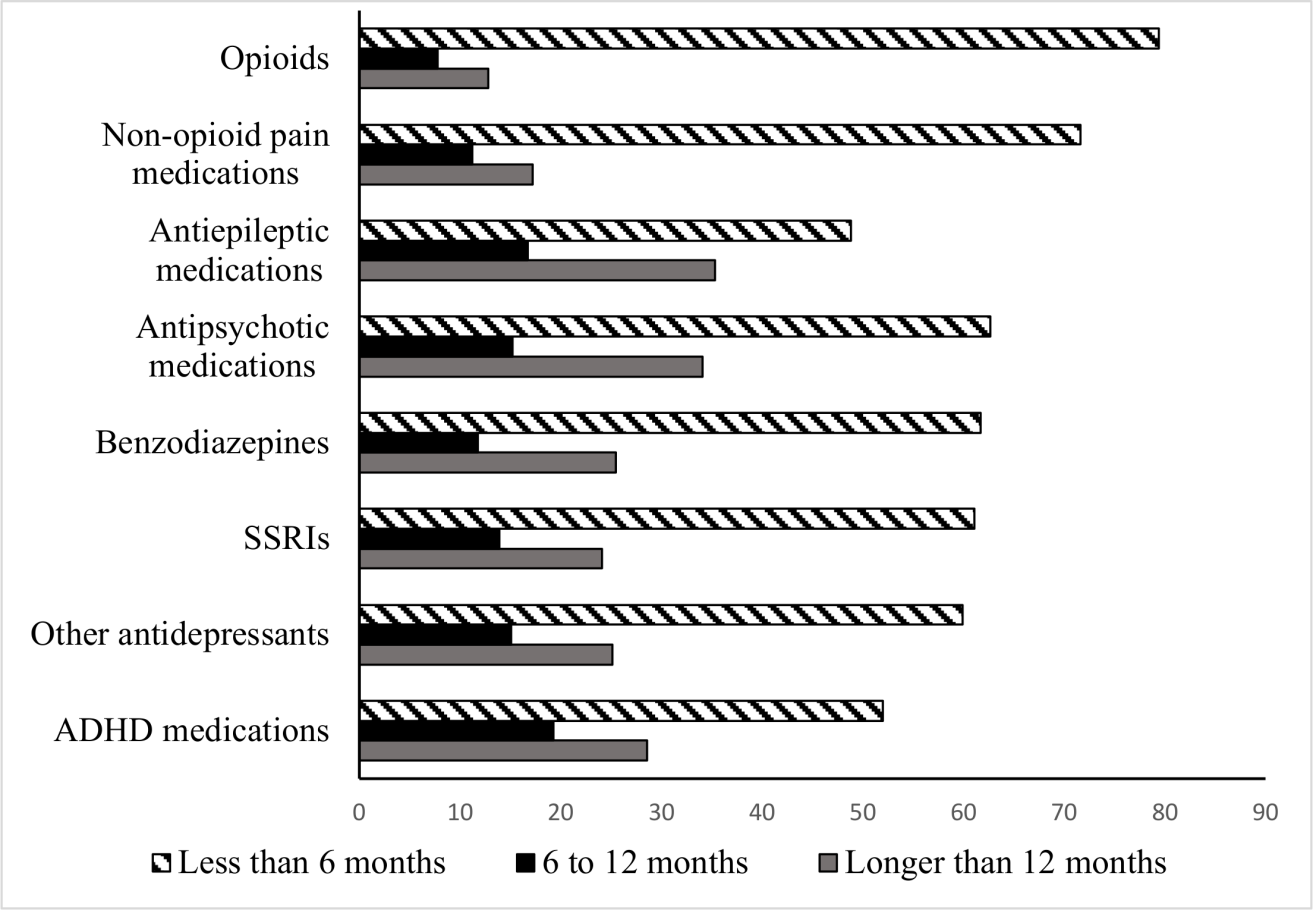
Duration of usage for psychotropic and pain medications prescribed during the 12 months after incident traumatic brain injury (TBI) (n=239 425). ADHD, attention-deficit hyperactivity disorder; SSRIs, selective serotonin reuptake inhibitors.

We examined the number of days between the first and last dispensed prescription within a medication period (defined as prescriptions collected less than 90 days apart) (figure 4). The majority of opioid medications were prescribed for less than 6 months, however, 20.6% of opioid prescriptions were prescribed over a period that exceeded 6 months. Non-opioid pain medications followed similar patterns. For benzodiazepines, although the majority of prescriptions were short-term: 37.3% of prescriptions were prescribed for 6 months or longer. For all other psychotropic medications, between 50% and 60% were prescribed for up to 6 months, and the remainder for periods longer than 6 months.

### Sensitivity analyses

We examined medication use by severity of the incident TBI by comparing individuals who received inpatient treatment to those receiving outpatient treatment of the incident TBI using logistic regression models (table 3). For the first 12 months post-TBI, individuals who had received inpatient treatment (27% of the cohort) demonstrated increased odds of any psychotropic and/or pain medication (OR: 2.40, 95% CI: 2.35 to 2.44), and of all medication classes, except for ADHD medications (OR: 0.91, 95% CI: 0.82 to 1.01), with the highest odds shown for opioids (OR: 2.08, 95% CI: 2.04 to 2.12) and non-opioid pain medications (OR: 2.68, 95% CI: 2.63 to 2.73). We also compared individuals with other TBIs to those with mild TBI only (table 3). We found no statistically significant associations for any medication use (OR: 0.99, 95% CI: 0.97 to 1.01). Individuals who had been treated for other TBIs demonstrated higher odds of benzodiazepines, antipsychotic, antiepileptic and non-opioid pain medications, while individuals with mild TBIs presented with higher odds of all other medications. Finally, we compared individuals with polytrauma (ie, at least one other co-occurring physical injury) to individuals with TBI without co-occurring physical injuries. Individuals with polytrauma presented with increased odds of any medication use (OR: 1.84, 95% CI: 1.80 to 1.88), and also of all medication classes except for antiepileptic medications. Highest ORs were found for opioids (OR: 2.39, 95% CI: 2.34 to 2.44) and non-opioid pain medications (OR: 2.13, 95% CI: 2.09 to 2.18).

**Table 3:** ORs of psychotropic and pain medications during the 12 months after incident traumatic brain injury (TBI) stratified by clinical characteristics

	Inpatient vs outpatient treatment OR (95% CI)	All other TBIs vs mild TBI OR (95% CI)	Polytrauma vs TBI without co-occurring physical injuries OR (95% CI)
Any psychotropic or pain medication	2.40 (2.35 to 2.44)	0.99 (0.97 to 1.01)*	1.84 (1.80 to 1.88)
Opioids	2.08 (2.04 to 2.12)	0.90 (0.89 to 0.92)	2.39 (2.34 to 2.44)
Non-opioid pain medications	2.68 (2.63 to 2.73)	1.09 (1.06 to 1.11)	2.13 (2.09 to 2.18)
Antiepileptic medications	1.73 (1.67 to 1.79)	1.16 (1.12 to 1.21)	0.97 (0.93 to 1.01)*
Antipsychotic medications	1.64 (1.58 to 1.71)	1.26 (1.20 to 1.31)	1.04 (0.99 to 1.09)*
Benzodiazepines	1.77 (1.73 to 1.82)	1.16 (1.12 to 1.19)	1.12 (1.09 to 1.16)
SSRIs	1.60 (1.56 to 1.64)	0.95 (0.93 to 0.98)	1.14 (1.11 to 1.17)
Other antidepressants	1.55 (1.50 to 1.59)	0.96 (0.93 to 0.99)†	1.15 (1.11 to 1.19)
ADHD medications	0.91 (0.82 to 1.01)*	0.73 (0.66 to 0.80)	1.18 (1.05 to 1.32)†

*†All differences were associated with p<0.0001, except for where indicated. *p>0.05. †p<0.05.

ADHD, attention-deficit hyperactivity disorder; SSRIs, selective serotonin reuptake inhibitors.

## Discussion

We have examined psychotropic and pain medication use in a nationwide cohort of 239 425 individuals diagnosed with TBI. We found that 37% of the cohort had been prescribed at least one psychotropic or pain medication in the 12 months before the incident TBI. In the 12 months after the incident TBI, there was an 8% increase in prescription of these medications, which meant that 45% of the cohort was prescribed one of these medications. Opioids and non-opioid pain medications were the most common medications, both before and after incident TBI. For the 12 months before the TBI, 16% were prescribed an opioid, and 20% were prescribed a non-opioid pain medication. For the 12 months after the TBI, rates increased to 22% and 27%, respectively. The two most common psychotropics were SSRIs (13%), and benzodiazepines (10%), which increased by around 1% after the TBI. Those with more severe injuries (as indicated by inpatient treatment or polytrauma) were around twice as likely to receive medications than those with less severe injuries.

Chronic pain is a common complication of TBI; a systematic review showed that almost 60% of TBI patients complained of headaches, and around 10% experienced other pain syndromes.^16^ This could be reflected in the increased rates of both opioid and non-opioid pain medication prescriptions after the TBI. Co-occurring physical injuries may further increase the risk of pain. Our results showed that individuals with polytrauma presented more than doubling of odds of opioid and non-opioid pain prescriptions, as compared with those treated for TBI without co-occurring physical injuries. On the other hand, 16% and 20% of the TBI cohort had been prescribed an opioid or non-opioid pain medication the 12 months preceding the TBI. Although opioids may provide an effective treatment for severe and chronic pain,^25^ they are associated with an increased risk of accidents,^26^ which could raise the risk of sustaining a TBI. Our results on post-TBI opioid use are similar to a study of US war veterans where a fifth initiated opioids after sustaining a TBI.^18^ However, 23% of the war veterans used for more than 12 months, as compared with 13% in our cohort. We found another 8% of our cohort were prescribed opioids for 6–12 months, which means that 21% were prescribed opioids for a longer-term. In addition to the contrasting background risks, co-occurring injuries, and possible TBI severity, one explanation for this difference could be higher levels of opioid prescriptions in the US than in Sweden (43 879 defined daily doses for statistical purposes per million Americans per day between 2011 and 2013 compared with 8343 in Swedes), which may be reflected in the duration of usage.^27^ Our results also showed that opioids were most commonly prescribed among individuals with post-TBI mood or anxiety disorders (around 35% respectively). Notably, 26% of individuals diagnosed with a substance use disorder were prescribed an opioid. Furthermore, individuals with more severe injuries (as indicated by inpatient treatment of the TBI or polytrauma) had more than twofold odds of using opioids when compared with those with less severe injuries (ie, outpatient treatment and TBI without co-occurring physical injuries, respectively). In contrast, individuals with mild TBI (ie, concussions) had 10% higher odds of using opioids. Little is known about the long-term consequences of opioid treatment in persons with TBI,^28^ however, opioids may increase the risk of cognitive and mental problems in persons with TBI.^29^ More studies on the long-term effect of opioids on TBI outcomes, as well as the factors that influence the initiation and continuation of opioid use among persons with TBI,^18^ are required. Alternative medications could be explored for pain management. Beta-blockers, antiepileptics and tricyclic antidepressants have been suggested for the prevention of migraine symptoms in TBI patients,^5^ however, there is little high-quality evidence on these medications to guide clinicians.^17 30^

We also found a high prevalence of medication use preceding the incident TBI—over one-third of the TBI cohort had been prescribed a psychotropic or pain medication in the 12 months leading up to the TBI. This proportion was considerably higher than the prevalence in unaffected siblings, who have similar genetic and early environmental backgrounds. Psychiatric and behavioural problems are known to increase the risk of sustaining a TBI,^31 32^ which could explain the higher prevalence (as compared with siblings) of both neuropsychiatric diagnoses and psychotropic medications before the TBI. Pre-TBI psychotropic medication was also increased in a study of US Medicare patients aged over 64.^10^ Thus, these previous investigations have suggested that the high medication use shown in other TBI cohorts^6–9^ may partly be attributed to prescriptions initiated before the injury.

We found that, after the incident TBI, absolute rates of any psychotropic or pain medication increased by around 8%. About half of the cohort (50%–60%) were prescribed psychotropic medications for less than 6 months after the TBI. We found that the most common psychotropic medication classes were antidepressants, consistent with previous pharmacoepidemiological studies in TBI samples.^6–10^ Around 10% of our cohort were prescribed benzodiazepines, both pre-TBI and post-TBI. Of those, 35% used them for more than 6 months after the TBI. Concerns about benzodiazepine use in individuals with TBI have been raised,^33^ as these medications may impair cognitive recovery and increase aggression,^5 34 35^ and long-term use is discouraged.^35^ Furthermore, we found that 22% of individuals with a substance use disorder diagnosis had been prescribed a benzodiazepine. Benzodiazepine use is associated with an increased risk of dependence and serious adverse effects, particularly among long-term users.^36^ There is therefore a need for more studies on the benefits and risks of long-term benzodiazepine use in individuals with TBI, particularly among individuals with comorbid substance use disorders.

The prevalence of medication use has varied considerably in previous work, possibly due to differing study populations and TBI severity.^6–10^ Prevalence of psychotropic medications (at 12 months post-TBI) was mostly lower in this TBI cohort than in prior research. This could be due to several reasons; national prescription practices may vary. Also, our cohort was population-based, and may differ in TBI severity, psychiatric history, comorbidities and age. However, when we stratified our sample by inpatient versus outpatient treatment, individuals who received inpatient management demonstrated higher odds of all psychotropic medication classes except ADHD medications, and prevalence rates of benzodiazepines, SSRIs and other antidepressants (online supplemental appendix table 3) were a similar to those reported in other work based in tertiary centres for postacute rehabilitation.^6 8^ However, when we stratified our sample by mild versus all other TBIs, there were no statistically significant differences in the use of any psychotropic and/or pain medication. Our cohort included individuals who were treated in hospitals or specialised outpatient care for their TBI, which could explain why there were only small differences in medication use between both groups. Nevertheless, our findings highlight the importance of routine follow-up after mild TBIs, including a review of psychotropic and pain medication prescriptions.

Furthermore, comedications were common—20% and 26% of the TBI cohort (before and after the TBI, respectively) were prescribed two or more medications within 12 months. The use of polypharmacy may reflect comorbidity of neuropsychiatric problems, as psychiatric conditions often co-occur.^37^ However, using multiple medications may exacerbate other health problems, and increase the risk of accidents, cognitive impairments and adverse drug–drug interactions.^38^ There is limited data on the effects of polypharmacy in TBI patients, and more studies are warranted.

### Strengths and limitations

This is, to the best of our knowledge, the first study to include a nationwide sample, with almost a quarter of a million individuals diagnosed with TBI. We linked several high-quality Swedish registers, and information on medications was based on individuals collecting their medication from pharmacies, which is an advance from prescription-only data, and was nearly complete (less than 0.3% missing information).^39^ For a comparison group, we included unaffected full siblings who have similar background characteristics (eg, family history and shared genetic and environmental factors). However, several limitations should be considered. Our analyses relied on collected prescriptions, which may or may not have been taken. Our information on neuropsychiatric diagnoses was collected from the Swedish Patient Register, which includes all disorders diagnosed in hospitals and specialised outpatient care, and therefore diagnoses solely made in primary care were not captured. We included only medications collected at pharmacies; medications used for treatment in hospitals are not available in the Swedish Prescribed Drug Register. For individuals with extended hospital stays, this could lead to underestimation of medication use, although in our cohort, the majority were not admitted overnight, and almost all were discharged within 30 days (99.8%, n=238 715). Moreover, differences between countries in prescription practices and service provision may affect the generalisability of findings. Sweden reports higher rates of TBI-related hospital discharges than the European average (age-adjusted rate per 100 000 individuals: Sweden 445.8; Europe 287.2), although this could in part be due to between-country differences in data collection and coding.^40^ The use of the Swedish Patient Register to identify individuals with TBI may involve selection effects and will likely underestimate TBI rates, particularly mild TBI. Finally, sibling controls were, on average, younger than the TBI cohort, but unlikely to explain differences in prescription rates.

## Conclusions

Our findings showed an elevated prevalence of psychotropic and pain medication dispensing in a population-based cohort of individuals who had sustained a TBI—both before and after the incident TBI. Opioids were prescribed to over 20% of the cohort after the TBI, with an absolute rate increase of around 5% compared with the 12 month period before sustaining a TBI. Although a majority were prescribed opioids for a short-term period, a fifth used them for more than 6 months. Similarly, over one-third of those prescribed benzodiazepines used them for longer than 6 months. To prevent complications associated with long-term opioid or benzodiazepine use, aftercare review of these medications should be routinely considered, including individuals with mild TBIs, and other treatment strategies should be encouraged.

## Data Availability

Data may be obtained from a third party and are not publicly available. The Public Access to Information and Secrecy Act in Sweden prohibits us from making individual level data publicly available. Researchers who are interested in replicating our work can apply for individual level data from: Statistics Sweden (mikrodata@scb.se) for data from the Total Population Register and the Multi-Generation Register; The National Board of Health and Welfare (registerservice@socialstyrelsen.se) for data from The Patient Register, The Prescribed Drug Register, and the Cause of Death Register.
