# Supplementary material for: Psychotropic and pain medication use in individuals with traumatic brain injury—a Swedish total population cohort study of 240 000 persons
Source: J Neurol Neurosurg Psychiatry. 2021 Feb 9;92(5):519–27. doi: 10.1136/jnnp-2020-324353 (PMC8053342; doi:10.1136/jnnp-2020-324353)
Supplement: Supplementary data [file jnnp-2020-324353supp001.pdf]

SUPPLEMENTARY APPENDIX TO THE MANUSCRIPT

Psychotropic and pain medication use in individuals with traumatic brain injury – A Swedish total population cohort study of 240,000 persons

Yasmina Molero, David J Sharp, Brian M D'Onofrio, Henrik Larsson, Seena Fazel

Contents

Appendix Methods………………………………………………………………………… 2
Participants and setting……………………………………………………………….. 2
TBI cohort ……………………………………………………………………………..2
Sibling controls……………………………………………………………………….. 2
Measures……………………………………………………………………………… 3
Medications…………………………………………………………………………… 3
Statistical analyses……………………………………………………………………. 3
Appendix Table 1…………………………………………………………………….... 4
Appendix Table 2…………………………………………………………………….... 5
Appendix Table 3…………………………………………………………………….... 6
References…………………………………………………………………………….. 7

APPENDIX METHODS

Participants and setting

TBI cohort

We used the Centers for Disease Control and Prevention definition of TBI[1] (International Classification of Diseases, 10th revision [ICD-10]: S01.0–S01.9, S02.0, S02.1, S02.3, S02.7–S02.9, S04.0, S06.0–S06.9, S07.0, S07.1, S07.8. S07.9, S09.7–S09.9, T01.0, T02.0, T04.0, T06.0, T90.1, T90.2, T90.4, T90.5, T90.8, T90.9). Individuals who had been diagnosed with TBI before July 2006 (ICD-9: 800-804, 851–854; ICD-10: as above) were excluded due to the data available on medications; we examined medication use 12 months prior to the TBI, and the Swedish Prescribed Drug Register started in July 2005.[2] Information on ICD-9/10 TBI diagnoses was collected from the Swedish Patient Register, which includes all admissions to hospitals and outpatient contacts with specialised secondary care.[3]. This register has excellent validity on ICD-9 concussion diagnoses (PPV= 100%; NPV= 99.8%),[4] however, other TBI diagnoses were not validated. The Swedish Patient Register also has excellent validity on inpatient treatment for ICD-10 TBIs (sensitivity= 95-97%; specificity=96-98%),[5] although diagnoses in outpatient treatment were not examined. Missing data in The Swedish Patient Register is around 1% for inpatient treatment, and around 3% for outpatient treatment.[3] Individuals who died within 7 days after the incident TBI (n=3,116) were not included in the cohort.

Sibling controls

Siblings were identified through the Multi-Generation Register,[6] and included all biological full siblings aged 18 and over who were alive and living in Sweden at the date of their sibling's TBI, and who had not been diagnosed with TBI (ICD-9/10) before December 31, 2013.

Measures

Medications

The Swedish Prescribed Drug Register includes information on all prescriptions that are collected from all Swedish pharmacies since July 2005, and has less than 0.3% missing information.[2] Medications included opioids (Anatomical Therapeutic Chemical [ATC] code: N02A), non-opioid pain medications (ATC: N02B), antiepileptic medications (ATC: N03), antipsychotic medications (ATC: N05A), benzodiazepines (ATC: N05BA), selective serotonin reuptake inhibitors (SSRIs; ATC: N06AB), other antidepressants (ATC: N06AA, N06AC, N06AF, N06AG, N06AX), and attention-deficit hyperactivity disorder (ADHD) medications (ATC: N06B).

Statistical analyses

We examined the duration of usage for prescriptions that were initiated within 12 months after the incident TBI. In these analyses, all prescriptions collected no more than 90 days apart were considered to belong to the same medication period, as The Swedish Pharmaceutical Benefits allows for a maximum of three months' supply for each prescription[7]. Prescriptions more than 90 days from the last prescription were considered to be the start of a new medication period. For individuals with more than one medication period within 12 months after the incident TBI, the longest medication period was chosen. We then calculated the number of days between the first and the last prescription within the medication period. All analyses were conducted using SAS version 9.4. We followed the Strengthening the Reporting of Observational studies in Epidemiology (STROBE) reporting guidelines.

Appendix Table 1. Number of individuals, χ^2, and degrees of freedom for Figure 1; medication use during the 12 months before and 12 months after incident traumatic brain injury (TBI).

	**TBI cohort - 12 months before incident TBI (n=239,425)**	**TBI cohort - 12 months after incident TBI (n=239,425)**	**Rate difference**	χ^2 **(*df*= 1)**
**Any psychotropic or pain medication**	36.6% (87,625)	45.0% (107,812)	8.4	81.4
**Psychotropic and pain medication classes**				
Opioids	16.3% (39,109)	21.6% (51,698)	5.3	46.1
Non-opioid pain medications	20.3% (48,559)	26.6% (63,710)	6.3	67.8
Antiepileptic medications	4.6% (11,086)	5.9% (14,129)	1.3	116.8
Antipsychotic medications	3.9% (9,258)	4.6% (11,033)	0.7	107.5
Benzodiazepines	10.1% (24,100)	11.3% (27,010)	1.2	94.8
SSRIs	12.6% (30,255)	13.5% (32,251)	0.9	127.7
Other antidepressants	6.8% (16,340)	8.0% (19,137)	1.2	96.4
ADHD medications	0.7% (1,552)	0.8% (1,822)	0.1	105.1
**Number of psychotropic and pain medication classes**				
One medication class	16.4% (39,256)	18.3% (43,786)	1.9	111.7†
Two and more medication classes	20.2% (48,369)	26.7% (64,026)	6.5	

Note: All rate differences were associated with p <.001. † $df = 4$

ADHD, attention-deficit hyperactivity disorder; SSRIs, selective serotonin reuptake inhibitors.

Appendix Table 2. Medication use in unaffected siblings during the 12 months before and 12 months after incident traumatic brain injury (TBI).

	**Siblings - 12 months before incident TBI (n=199,658)**	**Siblings - 12 months after incident TBI (n=199,658)**
**Psychotropic and pain medication classes**		
Opioids	8.3% (16,534)	8.6% (17,085)
Non-opioid pain medications	9.1% (18,116)	9.8% (19,547)
Antiepileptic medications	2.0% (3,914)	2.1% (4,159)
Antipsychotic medications	1.5% (2,964)	1.5% (3,045)
Benzodiazepines	3.6% (7,183)	3.7% (7,385)
SSRIs	5.8% (11,580)	6.0% (11,927)
Other antidepressants	3.7% (7,303)	3.8% (7,554)
ADHD medications	0.3% (638)	0.4% (747)

ADHD, attention-deficit hyperactivity disorder; SSRIs, selective serotonin reuptake inhibitors.

Appendix Table 3. Number of individuals for Table 3; Psychotropic and pain medications during the 12 months after incident traumatic brain injury (TBI), stratified by clinical characteristics

	**Outpatient treatment (n=174,648)**	**Inpatient treatment (n=64,777)**	**Mild TBI (n=68,381)**	**All other TBIs (n=171,044)**	**TBI without co-occurring physical injuries (n=194,916)**	**Polytrauma (n=44,509)**
**Any psychotropic or pain medication**						
	39.2% (68,456)	60.8% (39,356)	45.4% (31,015)	44.9% (76,797)	42.0% (81,943)	58.1% (25,869)
**Psychotropic and pain medication classes**						
Opioids	18.0% (31,411)	31.3% (20,287)	22.8% (15,613)	21.1% (36,085)	18.5% (36,050)	35.2% (15,648)
Non-opioid pain medications	21.0% (36,727)	41.7% (26,983)	25.5% (17,426)	27.1% (46,284)	23.6% (46,032)	39.7% (17,678)
Antiepileptic medications	5.0% (8,726)	8.3% (5,403)	5.3% (3,639)	6.1% (10,490)	5.9% (11,566)	5.8% (2,563)
Antipsychotic medications	4.0% (6,919)	6.4% (4,114)	3.9% (2,683)	4.9% (8,350)	4.6% (8,920)	4.8% (2,113)
Benzodiazepines	9.6% (16,757)	15.8% (10,253)	10.3% (7,027)	11.7% (19,983)	11.1% (21,555)	12.3% (5,455)
SSRIs	11.9% (20,774)	17.7% (11,477)	13.9% (9,502)	13.3% (22,749)	13.2% (25,689)	14.7% (6,562)
Other antidepressants	7.1% (12,332)	10.5% (6,805)	8.2% (5,612)	7.9% (13,525)	7.8% (15,196)	8.9% (3,941)
ADHD medications	0.8% (1,363)	0.7% (459)	0.9% (645)	0.7% (1,177)	0.7% (1,437)	0.9% (385)

ADHD, attention-deficit hyperactivity disorder; SSRIs, selective serotonin reuptake inhibitors.

REFERENCES

1. Coronado VG, Xu L, Basavaraju SV, et al. Surveillance for traumatic brain injury-related deaths--united states, 1997-2007. *MMWR Surveill Summ* 2011;60:1-32.
2. Wettermark B, Hammar N, Fored CM, et al. The new Swedish prescribed drug register--opportunities for pharmacoepidemiological research and experience from the first six months. *Pharmacoepidemiol Drug Saf* 2007;16:726-35.
3. Ludvigsson JF, Andersson E, Ekbom A, et al. External review and validation of the Swedish national inpatient register. *BMC Public Health* 2011;11:450.
4. Nilsson AC, Spetz CL, Carsjo K, et al. Reliability of the hospital registry. The diagnostic data are better than their reputation [Slutenvårdsregistrets tillförlitlighet. Diagnosuppgifterna bättre än sitt rykte]. *Läkartidningen* 1994;91:598, 603-5.
5. Gedeborg R, Engquist H, Berglund L, et al. Identification of incident injuries in hospital discharge registers. *Epidemiology* 2008;19:860-7.
6. Ekbom A. The Swedish multi-generation register. *Methods Mol Biol* 2011;675:215-20.
7. The Swedish Pharmaceutical Guidelines. Available at: www.fass.se 2018
